# Comparison of two reaction-time-based and one foraging-based behavioral approach-avoidance tasks in relation to interindividual differences and their reliability

**DOI:** 10.1038/s41598-023-49864-x

**Published:** 2023-12-16

**Authors:** Kim Fricke, Nina Alexander, Thomas Jacobsen, Susanne Vogel

**Affiliations:** 1 Department of Psychology, Medical School Hamburg, Am Kaiserkai 1, 20457 Hamburg, Germany; 2 Medical School Hamburg, ICAN Institute for Cognitive and Affective Neuroscience, Am Kaiserkai 1, 20457 Hamburg, Germany; 3 Department of Psychiatry and Psychotherapy, Philipps University Marburg, Rudolf-Bultmann-Str. 8, 35039 Marburg, Germany; 4 Experimental Psychology Unit, Helmut-Schmidt-University/University of the Federal Armed Forces Hamburg, Holstenhofweg 85, 22043 Hamburg, Germany

**Keywords:** Psychology, Human behaviour

## Abstract

Approaching rewards and avoiding punishments is a fundamental aspect of behavior, yet individuals differ in the extent of these behavioral tendencies. One popular method to assess differences in approach-avoidance tendencies and even modify them, is using behavioral tasks in which spontaneous responses to differently valenced stimuli are assessed (e.g., the visual joystick and the manikin task). Understanding whether these reaction-time-based tasks map onto the same underlying constructs, how they predict interindividual differences in theoretically related constructs and how reliable they are, seems vital to make informed judgements about current findings and future studies. In this preregistered study, 168 participants (81 self-identified men, 87 women) completed emotional face versions of these tasks as well as an alternative, foraging-based paradigm, the approach-avoidance-conflict task, and answered self-report questionnaires regarding anxiety, aggression, depressive symptoms, behavioral inhibition and activation. Importantly, approach-avoidance outcome measures of the two reaction-time-based tasks were unrelated with each other, showed little relation to self-reported interindividual differences and had subpar internal consistencies. In contrast, the approach-avoidance-conflict task was related to behavioral inhibition and aggression, and had good internal consistencies. Our study highlights the need for more research into optimizing behavioral approach-avoidance measures when using task-based approach-avoidance measures to assess interindividual differences.

## Introduction

Approach and avoidance of external stimuli are fundamental principles that shape an organism’s interaction with its environment^1^. At their core, these principles reflect the adaptive nature of behavior, as organisms strive to pursue rewards and positive consequences while simultaneously avoiding punishments and negative consequences. Gray and McNaughton^2^ proposed that three independent, but interacting, motivational systems are responsible for approach (behavioral activation system; BAS), avoidance (fight-flight-freeze system), and conflict resolution within and between the former two systems (behavioral inhibition system; BIS). In line with this theory, interindividual variability in approach-avoidance behaviors could then be explained by differences in any and all of these systems. Investigating those individual differences in approach-avoidance is important, as understanding approach-avoidance mechanisms better can, for example, provide insights into topics such as motivation, goal pursuit, risk-taking, or emotion regulation. Furthermore, many mental disorders are characterized by dysregulated approach-avoidance patterns. For example, individuals with anxiety disorders often exhibit excessive avoidance tendencies, where they actively avoid situations or stimuli they perceive as threatening or anxiety-provoking^3,4^. On the other hand, individuals with substance use disorders or pathological aggression may demonstrate excessive approach tendencies, while disregarding potential negative consequences^5,6^. Studying approach-avoidance tendencies could therefore aid in understanding the underlying mechanisms contributing to the development and maintenance of mental disorders.

**Figure 1:**
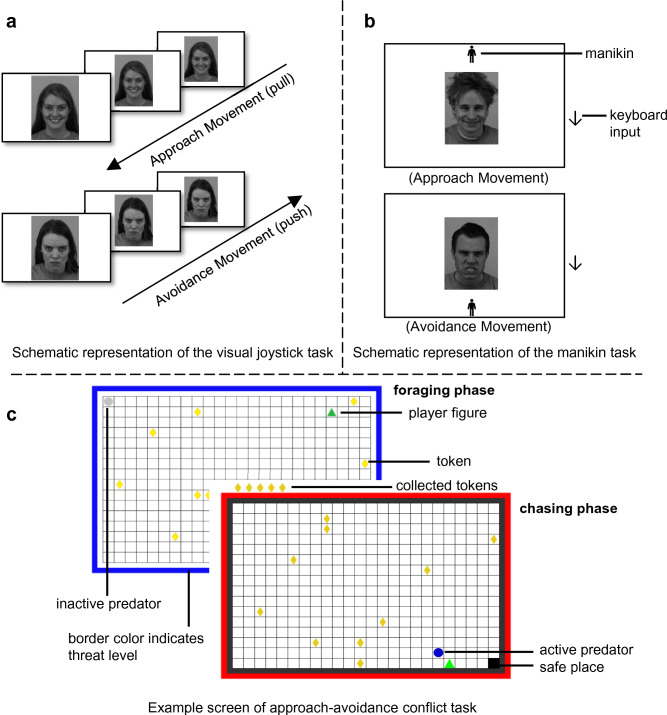
Examples of the three tasks, the (**a**) visual joystick, (**b**) manikin, and (**c**) AACT, utilized in this study. The representations of the visual joystick and manikin task are schematic and deviate in size and background color from the tasks performed by the participants. Greyscaled versions of the images with the codes AF21ANS, AF22HAS, AM28ANS and BM32HAS from Lundqvist, et al.^42^ are shown in the schematic representations of the visual joystick and manikin tasks and can be requested from kdef.se for non-commercial scientific research purposes. The figure has been adapted with permission from Fricke and Vogel^13^.

To investigate approach-avoidance tendencies in humans, there are several options including, for example, clinical assessment or interviews, natural or structured observations. Furthermore, self-report measures like questionnaires can be used to measure constructs related to approach and avoidance, for example, the behavioral inhibition (CW-BIS) and activation (CW-BAS) scales by Carver and White^7^, which aim to assess the sensitivities of Gray’s originally postulated general motivational systems^1^. Individuals with high CW-BIS-scores are believed to be highly sensitive to punishment and respond to potentially punishing stimuli with behavioral inhibition and increased anxiety, while participants with high CW-BAS-scores are highly sensitive to reward and actively approach potentially rewarding stimuli. To complement and extend these operationalizations of approach-avoidance constructs, reaction-time-based tasks have gained popularity as implicit measures of approach-avoidance tendencies, thus enabling the assessment of spontaneous responses to rewarding/punishing stimuli. Notably, the visual joystick task^8^ (adopted from Chen and Bargh^9^; see Fig. 1a) and the manikin task^10^ (see Fig. 1b) have emerged as prominent tasks for assessing approach-avoidance tendencies. In the visual joystick task, participants are instructed to push (avoid) or pull (approach) stimuli, resulting in a zoom effect, i.e., the shrinking or expanding of the respective stimuli. In the manikin task, instead, participants are instructed to direct a manikin towards or away from the stimulus. Instructions can either be directed at relevant (e.g., valence of the stimulus) or irrelevant (e.g., landscape vs. portrait presentation) stimulus features. In both tasks, approach-avoidance tendencies are quantified by the difference in response time for approaching and avoiding each stimulus, with faster avoidance indicating an avoidance bias and faster approach indicating an approach bias. To illustrate how the tasks are commonly structured, happy and angry faces, for example, are expected to elicit approach and avoidance biases, respectively. They are displayed in a congruent condition, where participants are instructed to approach happy and avoid angry faces, and subsequently in an incongruent condition with the instruction to avoid happy and approach angry faces. The reaction time differences between the congruent and incongruent condition within each stimulus category (happy, angry) would then represent the biases that are interpreted in terms of approach-avoidance tendencies.

While the visual joystick and manikin tasks are most commonly used, other attempts have been made to measure task-based approach-avoidance tendencies, for example, the approach-avoidance conflict task (AACT, see Fig. 1c) developed by Bach et al^11^., in which participants forage for tokens under threat. The AACT offers the advantage of being pharmacologically validated by demonstrating sensitivity to anxiolytics and comes with several outcome measures inspired by preclinical research, such as tracking the distance to the nearest wall or the predator. Importantly, the AACT differs from the visual joystick and manikin tasks in several ways. The visual joystick and manikin tasks introduce conflict implicitly by necessitating the over-riding of automatic tendencies in favor of goal-directed instrumental behavior, when instruction and stimulus content are incongruent, i.e., approaching negative and avoiding positive stimuli. This way, automatic biases are measured under time pressure (reaction-time-based tasks) with a clearly defined correct response. In contrast, the AACT can be understood as a more explicit measure that introduces direct goal conflicts in an ambiguous foraging scenario, i.e., the conflict between approaching tokens and avoiding predatory threat. In the AACT, there is no correct response, but rather different strategies to solve the goal conflict optimally based on individual preferences. Participants aim to maximize success, while being presented with potential positive and negative outcomes based on their decisions simultaneously. Inherent to all three tasks is that they induce some form of conflict and therefore are expected to activate Gray’s postulated approach-avoidance systems.

To facilitate understanding of approach and avoidance behavior, it is vital to investigate whether approach-avoidance measures of different tasks are comparable and map onto the same underlying constructs. Despite the widespread use of visual joystick and manikin tasks in research and clinical studies, we found only one study attempting to assess and compare their validity, showing no correlation between approach-avoidance biases of both tasks using spider and butterfly images with stimulus-relevant instructions, i.e., approaching or avoiding based on stimulus category, and thus indicating that they did not operationalize the same construct^12^. To the best of our knowledge, no studies to date have conducted direct task comparisons using emotional faces as stimuli. However, these comparisons appear relevant as emotional faces are widely used stimuli in research on, among others, aggression, anxiety, depression, trauma and stress within the approach-avoidance literature^13^. Thus, Aim 1 in this preregistered study was to investigate whether the outcome measures of emotional face versions of the visual joystick and manikin tasks are comparable operationalizations of the same underlying construct of approach-avoidance tendencies.

Next, as people observably differ in their approach-avoidance behaviors in real life, it seems further relevant to identify how well task-based measures can reproduce this interindividual variability. Based on the review by Fricke and Vogel^13^, criterion validity of approach-avoidance tasks seems not as consistent as anticipated, i.e., several interindividual differences did not reliably relate to task-based approach-avoidance measures. For instance, while several studies found links between anxiety and approach-avoidance behavior^14–19^; all joystick task studies had stimulus-irrelevant instructions, other studies did not^20–22^; two stimulus-irrelevant and one stimulus-relevant joystick task studies. Even in a large longitudinal study, Struijs et al^23^. found no association between task-based approach-avoidance tendencies of face stimuli (with stimulus-irrelevant instructions, i.e., to approach or avoid based on the color of the picture filter) and clinical anxiety or depression. These ambiguous findings were mirrored for several other interindividual differences^13^. For aggression, physical aggression has been linked to self-reports of the BAS^24^, while trait anger and psychopathy have been shown to elicit approach towards angry faces in approach-avoidance tasks^25^, but also not without some level of ambiguity^13^. We therefore aimed not only to compare the tasks, but also to assess their relationships with self-reported interindividual differences in behavioral inhibition and activation as well as trait anxiety and aggression, which are theoretically related to the stimuli we employed. Regarding Aim 2, we hypothesized that stronger avoidance biases should positively correlate with higher CW-BIS scores, whereas stronger approach biases should correlate with CW-BAS scores if task-based approach-avoidance biases are predictive for those interindividual differences. Likewise, more task-based avoidance should also be associated with more trait anxiety, while more task-based approach should be associated with trait aggression. For all hypotheses, influences of valence are expected, e.g., trait anxiety should be especially relevant in the context of angry faces. State anxiety and depressive symptoms were investigated in addition, the former expected to relate to increased avoidance of negatively valenced stimuli, while the latter was expected to relate to overall inhibition, i.e., slower overall reaction times, based on previous findings^26,27^. Regarding the BIS and anxiety, it is important to note that the concepts are strongly linked in the revised reinforcement sensitivity theory by Gray and McNaughton^2^. According to this theory, the detection of a goal conflict, i.e., an approach-avoidance, approach-approach or avoidance-avoidance conflict, will induce inhibition of current behavior and lead to an increase in state anxiety, while stable interindividual differences in BIS sensitivity should be related to observable differences in trait anxiety. This implies that BIS and trait anxiety self-report measures should correlate highly (as has for example been shown by Carver and White^7^) as both are most likely reflections of the self-registered frequency and intensity of anxiety as an emotional state. However, we think that the distinction between BIS and trait/state anxiety is still relevant to a degree as questionnaires based on the BIS conceptualization of the original reinforcement sensitivity theory may be reflective of both, the BIS and the fight-flight-freeze system^28^. To conclude, if the visual joystick and manikin task are differently suited to reflect these self-reported interindividual differences, the explained variance should differ between tasks. To offer perspective beyond the more established tasks, we also included the AACT as a less well-established paradigm. Previous research suggests a potential association between the AACT and trait anxiety and aggression^29^, although findings remain inconclusive as Bach et al^30^. found no link between a different measure of anxiety and AACT outcome measures in adolescents. We therefore extended Aim 2 to exploratively include the relationship of AACT outcome measures of approach and avoidance with the before mentioned self-reported interindividual differences.

In addition to our preregistered aims above and considering the underreporting of reliability measures in the literature, we also assessed internal consistencies for the outcome measures of all three tasks as Aim 3 of our study and hope this practice becomes more commonplace in future research. This appears especially relevant as clinical studies utilize approach-avoidance tasks as possible interventions to alter behavioral tendencies^31–33^, implying the need for consistent measurement tools that can reliably capture participants' approach-avoidance behaviors.

In summary, we used a correlational within-subject design in which participants completed three behavioral approach-avoidance tasks, namely the visual joystick, manikin, and AACT, and answered several personality questionnaires. This allowed us to compare the visual joystick and manikin tasks and whether they map onto the same underlying constructs (Aim 1), compare their ability to predict several self-reported interindividual differences (Aim 2) and report their internal consistencies (Aim 3). For Aim 2 and 3, the AACT, a pharmacologically validated task-based measure of approach-avoidance tendencies during foraging was also investigated. The hypotheses and analyses were preregistered at osf.io/ahvzx.

## Methods

### Participants

One hundred sixty-eight participants (81 self-identified men, 87 self-identified women, age: 18–56 years, mean: 22.85, SD: 4.90) with normal or corrected-to-normal vision and German as mother tongue or equivalent proficiency completed the study. A target sample size of at least 144 participants was supported by an a-priori power analysis (see preregistration at osf.io/ahvzx), allowing the discovery of medium-sized effects at an alpha error probability of 0.004 (alpha of 0.05 bonferroni-corrected for 12 planned comparisons) and a power of 80% for multiple linear regressions with four predictors (G*Power 3.1.9.7^34^; for details regarding the regression analyses, please refer to the “Statistical Analysis” section). Additional participants were tested due to being scheduled prior to reaching the target sample size. Participants provided written informed consent and student participants received partial course credit for participation. The study was approved by the local ethics committee (Ethikkommission der Medical School Hamburg, MSH-2019/79). All methods were performed in accordance with relevant guidelines, regulations and the Declaration of Helsinki.

### Experimental procedures

First, participants answered questionnaires assessing state and trait anxiety (STAI-S/T)^35^, trait aggression (DAF)^36^, depressive symptoms in the past two weeks (BDI-II)^37^, behavioral inhibition and approach (CW-BIS/BAS)^38^ as well as chronic stress in the past three months (TICS; not reported here)^39^. This was followed by the three behavioral tasks (visual joystick, manikin, and AACT) in counterbalanced order with short breaks in-between. Afterwards, participants answered Likert scale questions regarding drug use, gaming habits and current physical and psychological strain (not reported here). In the end, participants were debriefed about study procedures and informed about psychological help services in case of heightened depressive symptomatology (BDI-II ≥ 20 or indicated suicidality). The experiment lasted approximately 100 min (SD: 13 min).

### Visual joystick and manikin task

The visual joystick and manikin task were adapted from Inquisit 5 templates^40,41^. To enhance comparability, task designs were aligned as much as possible.

#### Stimulus material

Emotional face images from the Karolinska Directed Emotional Faces^42^ (size: 562 × 762 px) and Radboud Faces Database^43^ (size: 681 × 1024 px) were gray-scaled and rated based on their emotional valence, intensity and credibility of expression by three independent raters. Ninety-six images with angry/happy expressions of male/female faces (24 images each) were selected and divided evenly across the two tasks (see Supplementary Information).

#### Task design

Following 8 (manikin task) and 10 (visual joystick task) practice trials, participants performed 16 blocks of 12 trials (192 trials total) per task. Participants were instructed to either approach or avoid stimuli presented in the middle of a 22″ computer screen as follows: Approach was implemented by pulling a joystick towards the participant (visual joystick task, Fig. 1A) or by moving a manikin towards the stimulus by pressing the up (8-)key or down (2-)key on the num keyboard depending on the manikin’s location (manikin task, Fig. 1B). In contrast, participants were instructed to avoid by pushing a joystick away from the participant or moving the manikin away from the stimulus. In the visual joystick task, approach and avoidance were visually enhanced by real-time (i.e., proportional to the speed of the joystick movement) zoom-in/zoom-out effects of the stimulus on a white background. Initially, stimuli were scaled to fill 60% of the screen height and could be pushed to fill 10% or pulled to fill 100% of the screen height before disappearing. In the manikin task, animations of the manikin walking towards/away of the stimulus, which filled 40% of screen height on a black background, indicated approach and avoidance. Instructions varied block-wise between approach of happy/avoidance of angry faces (congruent) and approach of angry/avoidance of happy faces (incongruent). Congruency conditions of the first block were counterbalanced across participants and then switched after each block. Presentation order of stimuli was pseudo-randomized with all 48 stimuli within a task appearing twice per instruction with the limitations of only three stimuli in a row having the same valence or gender and stimuli only repeating after all 48 stimuli were displayed per instruction. In addition, the manikin appeared both above and below each stimulus once per instruction of the manikin task.

#### Indices (outcome parameters)

We recorded response correctness and reaction times (RT) for correct responses (visual joystick task: full extension of the joystick in correct direction; manikin task: press of 8- or 2-key on numpad). For RTs, values below 200 ms and above 1,500 ms were discarded. Values exceeding 3 SDs or more above/below the individual mean (of each specific valence/instruction combination) were subsequently removed. Moreover, data from two participants in the visual joystick and nine participants in the manikin task was excluded as more than 25% of trials were removed through the above steps. To ensure that post-error slowing in the visual joystick and manikin tasks had no effect on the respective association with outcome measures, we included the option to run the analysis with post-error-trials excluded in our analysis file (osf.io/ahvzx) as suggested by a reviewer. However, the results did not differ substantially from our main analysis.

Next, we constructed mean RT scores per task for all combinations of stimulus valence (happy/angry) and instruction (approach/avoid) as well as an overall mean RT score. Bias scores were then constructed by subtracting mean RTs of avoidance trials from approach trials per valence category. For example, the bias score for happy faces is the mean RT to make an approach movement towards happy faces minus the mean RT to make an avoidance movement away from happy faces. A negative score thus indicates faster approach, while a positive score indicates faster avoidance. Following reviewer suggestion, we additionally included a valence-unspecific bias measure as this is more in line with the phrasing of our hypotheses regarding general approach and avoidance biases. Moreover, we also included a global congruency measure in which reaction times in congruent trials, i.e., approaching happy and avoiding angry faces, were subtracted from reaction times in incongruent trials, i.e., avoiding happy and approaching angry faces.

In addition, so called D-Scores^44^ were constructed for exploratory analyses as these are reportedly better suited measures of RT differences^45^. To construct these, the differences of mean RTs are divided by their pooled standard deviation (irrespective of instruction) and data exclusion takes error rates into account by replacing error trials by the block RT mean + 2 SD (irrespective of stimulus valance).

### Approach-avoidance conflict task

The AACT was adapted from Bach et al^11^. in Python 3.2.5 using Pygame 1.9.2 code available from osf.io/d69pr^46^.

#### Task design

For 160 trials (divided into four blocks), participants were instructed to forage for tokens on a 24 × 16 grid containing ten tokens in variable locations, a predator in one corner, and a safe space in the opposite corner. Avoidance motivation was induced by threat of the predator waking up, chasing participants and taking away the in-trial earned tokens (see Fig. 1C). Threat level was manipulated by having high vs. low threat predator conditions (half of trials each) based on wake-up probability (60 vs. 20%). Initial threat distance was manipulated by placing participants either by the predator or the safe space (half of trials each). Trials lasted between 6 and 15 s and were extended by a 3.5 s chase-phase in case of predator wake-up. There was no monetary incentive for token collection. After task completion, participants estimated the predator wake-up probabilities. For a detailed description of the AACT, see the supplement of Fricke et al^46^.

#### Indices (outcome parameters)

We selected ten previously established outcome parameters^11,29,47^. Three measures were per-trial measures (recorded once per trial): Foraging latency, i.e., time until the first button press, as measure of initial decision processes; sum of tokens retained (unless caught) to measure overall performance; and failure to avoid threat (i.e., whether the participant got caught) as additional performance measure. Seven measures were in-trial measures (recorded every 500 ms): distance to closest wall, presence in safe quadrant of the board (12 × 8 field in which the safe place was located), presence in safe place, presence in dangerous quadrant of the board (12 × 8 field in which the predator was initially located), distance to predator, rate of token collection, and running speed on grid. All measures were averaged over trials (and time points for in-trial measures). As per reviewer’s suggestion, we additionally increased resolution by separating the outcome parameters by initial threat distance (close, far) and threat level (low, high), leading to an additional 40 outcome parameters which were investigated in exploratory analyses that paralleled the statistical analyses regarding Aim 2 in the supplement (see supplementary section Approach-Avoidance Conflict Task analyses including threat level and threat distance as variables). These analyses were initially not included as a prior study reported outcome measure interactions with threat level to be rather unreliable and that behavior became comparable over time for close and far initial threat distance^30^.

### Statistical analysis

All analyses were conducted in R (Version 4.3.1) and can be found, accompanied by the data, at osf.io/ahvzx. To compare the visual joystick and manikin task (Aim 1), we constructed Pearson correlation coefficients for all outcome measures (overall RT, overall accuracy, global congruency, approach-avoidance bias scores and exploratively D-Scores) of the visual joystick task with the respective outcome measure of the manikin task to investigate their similarities and differences.

To answer our main preregistered hypotheses, investigating the ability of all three tasks to predict self-reported interindividual differences (Aim 2), we first provided a correlation matrix (with probabilities not corrected for multiple comparisons) of all task outcome measures (of visual joystick, manikin, and AACT) with all questionnaire scores (CW-BIS/BAS, STAI-S/T, DAF subscales physical aggression, verbal aggression, and anger, and BDI) as an overview. Then, we constructed multiple linear regression models to investigate how predictive the approach-avoidance tasks were of the personality variables measured by questionnaires. For both visual joystick and manikin task, we used the predictors overall RT, overall accuracy, valenced approach bias and avoidance bias scores as well as the control variables age and gender. The models were then compared to their respective baseline models containing only age and gender by testing whether the model fit was significantly improved. We tested for the assumption of independence with the Durbin-Watson-Test accepting values between one and three and for the assumption of no multicollinearity by variance inflation factors (VIF), checking whether the largest VIF would exceed a value of ten or the average VIF substantially exceeded a value of one^48^. If studentized residuals were distributed non-normally as tested with a Shapiro–Wilk test, bias-corrected and accelerated bootstrapped confidence intervals based on 2,000 bootstrap repetitions are reported. The same procedure was then repeated for an alternative model in which potentially influential cases with standardized residuals exceeding two standard deviations away from the mean were excluded on the basis of Cook’s distance exceeding a value of one, leverage values three times larger than the average leverage and/or the covariance ratio falling outside of one plus/minus three times the average leverage. In case of meaningful differences in models, i.e., only one of the models being a significantly better fit than the respective baseline model or predictors in models differing in their significance, findings of both models are reported. Outcome variables of the regression models were the CW-BIS/BAS, STAI-T and DAF (subscales: physical aggression; verbal aggression; anger) scores. Given the two tasks, this resulted in 12 multiple linear regression models that were corrected for multiple comparisons with the Benjamini–Hochberg procedure in line with our preregistered analysis strategy. The correction was separately applied for the standard model and the model with removed influential cases. In addition, exploratory models were constructed for STAI-S and BDI scores. These were corrected for multiple comparisons as the original 12 models by removing the four highest p-values of those 12 models and combining the remaining eight p-values with the four p-values of the STAI-S and BDI models. Following reviewer suggestion, we repeated the regression analyses with the additional predictors valence-unspecific bias and global congruency. If the inclusion of the additional predictors resulted in more predictive models over the control models, we report the findings in addition to the original regression analyses.

Exploratively, we also constructed multiple regression models for the AACT as described for the other two tasks with the following changes: Since the ten predictor variables, i.e., the outcome measures of the AACT, had VIFs exceeding values of ten, we opted for parameter selection via least absolute shrinkage and selection operator regression before testing the selected parameters in the regression models described above. Age and gender were not included in the parameter selection, but added to the full models, which were compared against their respective baseline models containing only age and gender. We are aware that selecting variables that explain the most variance before for the regression models biases our data towards significant findings. Therefore, any findings should be interpreted with caution and tested with confirmatory hypotheses in the future. For the AACT, six models (Benjamini–Hochberg corrected) were generated for the outcome variables CW-BIS/BAS scores, STAI-T score and DAF-subscales physical aggression, verbal aggression and anger. In addition, exploratory models for STAI-S and BDI scores were corrected at the level of the original six models by removal of the two highest *p*-values.

To address Aim 3, we investigated internal consistencies of all task measures by separating trials based on task-factors first (visual joystick/manikin task: stimulus valance x instruction to approach or avoid; AACT: threat level x threat distance [x time in trial for in-trial variables, which were assessed every 500 ms]) and then splitting them in odd and even trials. We then averaged outcome measures per participant and calculated Pearson correlation coefficients for the aggregated odd and even values. We permuted our data set one thousand times (with the constraint of only permuting within block for visual joystick and manikin task) and repeated the procedure. Correlation coefficients were then averaged and 95% confidence intervals constructed based on the 25th and 975th highest correlation coefficient. Due to the task being split in half, Spearman-Brown prophecy formula-corrected consistencies were also reported. Similarly, we constructed split-half internal consistencies and Spearman-Brown prophecy formula-corrected consistencies for all investigated questionnaires (or subscales). All reported p-values of our analyses are tested for significance at an alpha-level of 0.05.

In our preregistration, we intended to compare models across tasks by their Akaike Information Criterion^49^. Given that task outcome parameters in our models were rarely influential, witnessed by no better model performance than the respective baseline model, we did not pursue direct comparisons of the three tasks. We also did not pursue an exploratory factor analysis, preregistered as exploratory analysis, across all task outcome measures to find commonalities between the tasks more directly due to an inadequate Kaiser–Meyer–Olkin factor^50^ that could not be resolved without the exclusion of too many of our task outcome variables.

## Results

In the visual joystick task, participants averaged overall RT scores of 755 ms (SD: 78 ms) with an accuracy of 95% (SD: 4%). As expected, average bias scores indicated tendencies to approach happy faces (-36 ms) and avoid angry faces (2 ms, difference: t_165_ = -5.78, p < 0.001). The D-Scores showed a similar trend of happy face approach (− 0.25) and angry face avoidance (0.023, difference: t_165_ = − 6.20, *p* < 0.001). An ANOVA with valence (happy, angry) and instruction (approach, avoid) as within-subject factors, i.e., including the global congruency effect, showed a significant interaction of valence and instruction (F_1165_ = 37.89, *p* < 0.001, η^2^G = 0.014), which was qualified post hoc by an approach bias towards happy faces (F_1165_ = 59.10, *p* < 0.001, η^2^G = 0.043), but no significant avoidance bias of angry faces.

In the manikin task, participants averaged overall RT scores of 739 ms (SD: 90 ms) with an accuracy of 94% (SD: 4%). As in the visual joystick task, bias scores and D-Scores could be differentiated based on stimulus category (bias–happy faces: − 108 ms, angry faces: − 63 ms, difference: t_158_ = − 7.69, *p* < 0.001; D-Score–happy faces: − 0.55, angry faces: − 0.32, difference: t_158_ = − 7.42, *p* < 0.001) and indicated stronger approach for happy than angry faces. A valence x instruction ANOVA showed a significant interaction (F_1158_ = 57.65, *p* < 0.001, η^2^G = 0.014), which was qualified post hoc by approach biases towards happy (F_1158_ = 478, *p* < 0.001, η^2^G = 0.224) and to a lesser degree angry faces (F_1158_ = 182, *p* < 0.001, η^2^G = 0.082).

**Figure 2:**
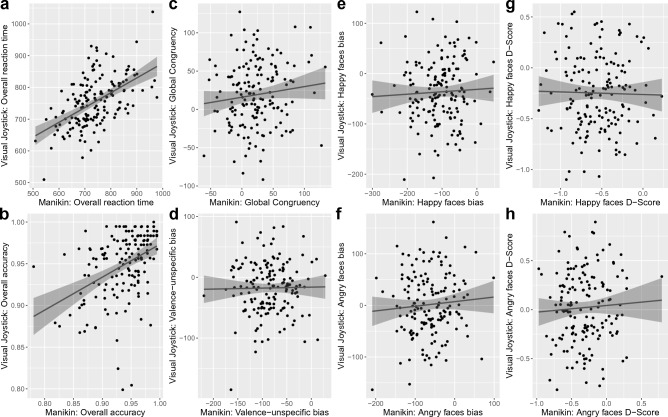
Correlations of visual joystick and manikin task measures (**a**) overall reaction time (r_156_ = .53, *p* < .001), (**b**) overall accuracy (r_156_ = .41, *p* < .001), (**c**) global congruency (r_156_ = .12, *p* = .135), (**d**) valence-unspecific bias (r_156_ = .02, *p* = .844), (**e**) happy faces bias (r_156_ = .05, *p* = .551), (**f**) angry faces bias (r_156_ = .08, *p* = .339), (**g**) happy faces D-Score (r_156_ = -.02, *p* = .796) and (**h**) angry faces D-Score (r_156_ = .05, *p* = .495). Line indicates linear regression over all data points with 95% confidence interval.

Regarding Aim 1, the comparison of visual joystick and manikin task outcome measures revealed no correlations between bias and D-Scores of the two tasks, while overall reaction time (r_156_ = 0.53, p < 0.001) and accuracy (r_156_ = 0.41, p < 0.001) were moderately correlated (see Fig. 2). Participants were therefore comparably fast and accurate in both tasks, but bias and D-Scores were unrelated, indicating that the tasks are not comparable operationalizations of the same underlying construct despite ostensibly similar measures of approach and avoidance.

**Figure 3:**
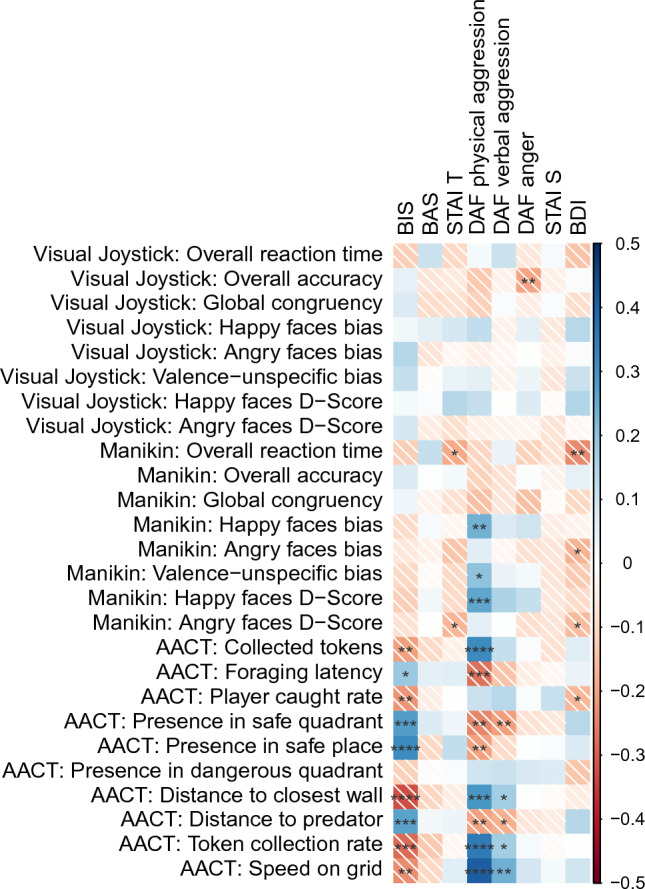
Correlation matrix of all questionnaire scores with task outcome measures. Color intensity indicates strength of correlation, color and shade indicate direction of effect (blue/no shade = positively correlated; red/ shaded = negatively correlated). *p*-values are uncorrected for multiple comparisons. Significance codes: **** *p* < .0001, *** *p* < .001, ** *p* < .01, * *p* < .05.

Concerning interindividual differences, the sample showed sufficient variance for all self-reported questionnaire scores (see Table 1; for boxplots/density plots, see Supplementary Figure S1). Compared to (non-clinical) norm samples, values were normal to slightly elevated for trait anxiety and depressive symptoms. CW-BIS and CW-BAS were comparable to the published norm^38^. Internal consistencies of the questionnaires were mostly acceptable with only CW-BAS and verbal aggression seemingly subpar (see Table 1). Figure 3 shows the correlation matrix (uncorrected probabilities) of all task outcome measures with all questionnaire scores (Aim 2). In contrast to our hypotheses, bias and D-Scores of the visual joystick task were not significantly correlated with any self-reported questionnaire scores. In the manikin task, physical aggression was related to stronger avoidance of happy faces (bias: r_157_ = 0.24, *p* = 0.003; D-Score: r_157_ = 0.26, *p* < 0.001) and valence-unspecific avoidance (r_157_ = 0.20, *p* = 0.010), whereas trait anxiety (D-Score: r_157_ = − 0.17, *p* = 0.035) and depressive symptoms (bias: r_155_ = − 0.17, *p* = 0.029; D-Score: r_155_ = − 0.16, *p* = 0.049) related to the approach of angry faces. Higher depressive symptom scores (r_155_ = − 0.24, *p* = 0.003) and trait anxiety (r_157_ = − 0.19, *p* = 0.017) were additionally associated with significantly faster overall reaction times. These findings regarding the visual joystick task and the manikin task were not in line with our hypotheses.

**Table 1:** Mean, standard deviation, range and internal consistency estimates of all self-reported measures per gender.

Questionnaire cale	Men		Women		Internal consistency estimate (SB)
	Mean (sd)	Range (min; max)	Mean (sd)	Range (min; max)	
CW-BIS	2.73 (0.53)	2.57 (1.14; 3.71)	3.16 (0.56)	2.29 (1.71; 4)	0.69 (0.82)
CW-BAS	3.15 (0.37)	1.85 (2.15; 4)	3.15 (0.35)	1.61 (2.31; 3.92)	0.56 (0.72)
STAI S	36.28 (6.49)	31 (22; 53)	36.51 (7.52)	48 (23; 71)	0.75 (0.85)
STAI T	38.69 (9.15)	46 (22; 68)	41.44 (10.91)	44 (24; 68)	0.85 (0.92)
DAF physical aggression	15.53 (5.1)	20 (9; 29)	11.7 (3.63)	20 (9; 29)	0.76 (0.86)
DAF verbal aggression	11.28 (2.42)	12 (7; 19)	10.32 (2.31)	11 (6; 17)	0.40 (0.58)
DAF anger	12.99 (3.66)	18 (7; 25)	12.63 (4.13)	17 (7; 24)	0.68 (0.81)
BDI	8.67 (6.99)	39 (0; 39)	10.47 (8.28)	35 (0; 35)	0.81 (0.90)

SB = Spearman-Brown-corrected.

For the AACT, plausible clusters of correlations emerged for the BIS, physical aggression and, to a lesser degree, verbal aggression. A higher CW-BIS score was related to more cautious behavior, more precisely, initiating foraging later (r_166_ = 0.19, *p* = 0.016), collecting fewer tokens (total tokens: r_166_ = − 0.21, *p* = 0.007; collection rate: r_166_ = − 0.28, *p* < 0.001), being caught less frequently by the predator (r_166_ = − 0.22, *p* = 0.004), staying more in safe areas (safe quadrant: r_166_ = 0.28, *p* < 0.001; safe place: r_166_ = 0.32, *p* < 0.001) and therefore farther away from the predator (r_166_ = 0.27, *p* < 0.001), staying closer to the walls (r_166_ = − 0.32, *p* < 0.001), and moving generally slower throughout the task (r_166_ = − 0.23, *p* = 0.002). The opposite pattern was true for physical and verbal aggression, which led to faster initiation of foraging (physical: r_166_ = − 0.27, *p* < 0.001), collecting more tokens (physical: total tokens: r_166_ = 0.32, *p* < 0.001; collection rate: r_166_ = 0.41, *p* < 0.001; verbal: collection rate: r_166_ = 0.24, *p* = 0.024), staying more outside safe areas (physical: safe quadrant: r_166_ = − 0.24, *p* = 0.001; safe place: r_166_ = − 0.20, *p* = 0.008; verbal: safe quadrant: r_166_ = − 0.21, *p* = 0.007), closer to the predator (physical: r_166_ = − 0.20, p = 0.008; verbal: r_166_ = − 0.18, *p* = 0.020), further away from the walls (physical: r_166_ = 0.29, *p* < 0.001; verbal: r_166_ = 0.17, *p* = 0.023) and moving generally faster during the task (physical: r_166_ = 0.41, *p* < 0.001; verbal: r_166_ = 0.24, *p* = 0.001). These clusters in the AACT convincingly indicate more approach tendencies in more aggressive individuals and more avoidance tendencies in participants with stronger behavioral inhibition, which was in line with theoretical expectations.

Regression models including visual joystick and manikin task outcome measures had no incremental predictive value for any investigated questionnaire scores beyond age and gender alone (*p* > 0.099). This was also the case for models including the additional predictors valence-unspecific bias and global congruency. In contrast, AACT regression models predicting BIS, BAS, physical aggression, verbal aggression and depressive symptom scores performed significantly better than their respective baseline models including only age and gender (see Table 2 for a list of the significant models and their predictors). In particular, less presence in the safe place was predictive of higher CW-BAS scores and higher speed on grid predictive of higher physical aggression and depressive symptom scores. Gender was a significant predictor for several models despite being also included in the respective baseline models with women scoring higher in BIS and depressive symptoms, but lower in physical aggression. It is important to keep in mind that these regression models on AACT data warrant independent replication as they were constructed based on preselected predictors explaining the most variance to reduce multicollinearity, thereby increasing the likelihood of significant findings.

**Table 2:** Significantly better multiple regression models of the AACT compared to baseline models (with predictors).

Questionnaire	Predictor variables	Original model	Alternative model
		Model fit/sig. β estimates	Model fit/sig. β estimates
CW-BIS			adj. R^2^ = .20, F(6,156) = 7.772, *p* < .001
	**Gender**		0.410***
	Age		–
	Presence in safe quadrant		–
	Presence in safe place		–
	Distance to closest wall		–
	Token Collection Rate		–
CW-BAS		adj. R^2^ = .06, F(7,160) = 2.534, *p* = .017	adj. R^2^ = .08, F(7,154) = 2.920, *p* = .007
	Gender	–	–
	Age	–	–
	Player caught rate	–	–
	**Presence in safe place**	-4.400***	-4.574***
	Presence in dangerous quadrant	–	–
	Distance to closest wall	–	–
	Token Collection Rate	–	–
Physical aggression		adj. R^2^ = .19, F(4,163) = 10.970, *p* < .001	adj. R^2^ = .25, F(4,157) = 14.070, *p* < .001
	**Gender**	-2.260*^a^	-2.622**
	Age	–	–
	Presence in safe quadrant	–	–
	**Speed on grid**	2.716*	2.041*^a^
Verbal aggression			adj. R^2^ = .08, F(4,159) = 4.510, *p* = .002
	Gender		–
	Age		–
	Presence in safe quadrant		–
	Speed on grid		–
BDI		adj. R^2^ = .08, F(6,159) = 3.541, *p* = .003	adj. R^2^ = .06, F(6,153) = 2.650, *p* = .018
	**Gender**	3.414*^a^	–
	Age	–	–
	Foraging latency	–	–
	Player caught rate	–	–
	Presence in safe quadrant	–	–
	**Speed on grid**	8.043**	5.435**

Alternative model: Model without cases with standardized residuals exceeding two standard deviations away from the mean and Cook’s distance exceeding a value of one, leverage values three times larger than the average leverage and/or a covariance ratio outside of one plus/minus three times the average leverage. Significant predictors are in bold. ^a^Not significant based on bootstrapped confidence interval. Significance codes: *** *p* < 0.001, ** *p* < 0.01, * *p* < 0.05.

**Figure 4:**
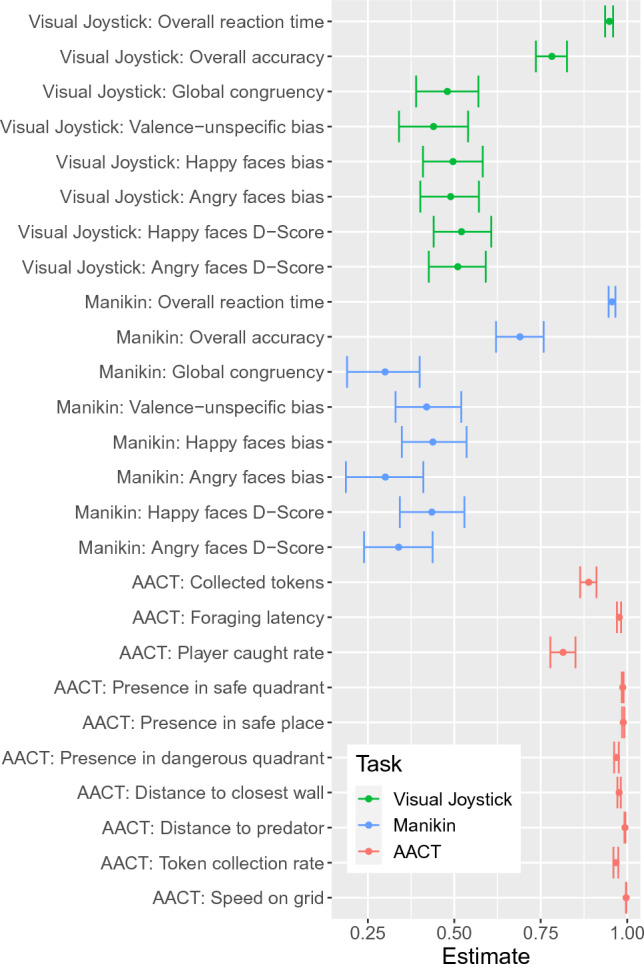
Internal consistency estimates for all outcome measures sorted by task. Bars indicate the confidence interval based on the 25th and 975th value based on thousand randomly permutated datasets (for restrictions see “Methods” section).

Finally, and regarding Aim 3, the internal consistencies of all visual joystick and manikin task approach-avoidance measures (global congruency, bias and D-Scores) were subpar (all r ≤ 0.52/Spearman-Brown-corrected (SB): r ≤ 0.69), indicating low consistency for both tasks (see Fig. 4). Only the consistency of general performance measures was good (overall reaction time (r ≥ 0.95/SB: r ≥ 0.97); accuracy (r ≥ 0.69/SB: r ≥ 0.82), but these are not specific to approach-avoidance. Internal consistencies for the AACT can be considered good to excellent (all r ≥ 0.81/SB: r ≥ 0.90), indicating that participants behaved consistently throughout the task, a finding that was already suggested by Bach et al^11^.

## Discussion

Approach-avoidance tasks, such as the visual joystick and manikin tasks, are one way to operationalize approach-avoidance tendencies that are widely used in research. However, although these reaction-time-based tasks aim to measure the same underlying constructs, it remains unclear whether they are actually comparable. Additionally, concerns have been raised regarding the ability of these tasks to predict interindividual differences which are theoretically strongly associated with approach-avoidance tendencies^13^. To address these issues, the present study sought to compare proposed indices of approach-avoidance tendencies between the visual joystick and manikin tasks (Aim 1). Furthermore, the explanatory power of both tasks and the AACT, a pharmacologically validated approach-avoidance task developed in the context of anxiety, were assessed for several self-report measures, which are theoretically related to approach and avoidance (Aim 2). Internal consistencies of the tasks were also examined as reliability measures of the tasks are seldom given, but relevant, for example, in interindividual differences research and the context of clinical studies (Aim 3).

### Visual joystick and manikin tasks: Convergent validity and association with self-report questionnaires

The bias measures of the visual joystick and manikin tasks, supposedly indexing approach-avoidance tendencies, were not correlated with each other. This lack of association is surprising as the sample was sufficiently large and heterogenous, the task designs of both tasks comparable (e.g., stimuli, trial number, blocks), the outcome measures analogue, and the statistical analyses identical. One potential explanation for the missing correlations could be that the tasks differed in their salience. The visual joystick task adopts a self-related frame of reference, i.e., pulling and pushing towards/away from oneself, and includes real time zoom-in/-out effects of the stimuli, which prevent recategorizing of the joystick movements^51^. In comparison, in the manikin task approach-avoidance behavior was more representational by moving a manikin toward/away from the stimulus per button press and receiving visual feedback only after the response is made. Due to the different framing, the manikin task may appear considerably less salient and therefore have introduced less approach-avoidance conflict in the incongruent condition. However, even with differences in saliency, associations beyond general performance measures would have been expected if both tasks measure the same underlying constructs. In addition, the potential increase in saliency in the visual joystick task did not translate into stronger associations with self-report measures (see below) rendering interpretations difficult. Saliency might have been especially lacking for angry face stimuli, which did not elicit avoidance biases in either task averaged across participants. Nevertheless, it is important to note that the commonly examined outcome measures in the existing literature primarily focus on the differences in bias between distinct stimulus categories (e.g., happy vs. angry faces), which were indeed present in our data. Moreover, the absence of avoidance bias on group level does not negate that variance in approach-avoidance tendencies between participants should have led to between-task associations if the measures were related. We conclude that, at least for the emotional facial stimuli presented here, the tasks’ bias measures do not assess the same underlying constructs of approach and avoidance, which warrants further investigation in the future.

In regard to self-reported measures of interindividual differences, outcome measures of the visual joystick task did not explain any significant variability. For the manikin task, correlations indicated avoidance tendencies for happy faces in participants with higher physical aggression and approach tendencies for angry faces in trait anxious and depressive individuals, which seems hard to reconcile with theoretical accounts^3,5^. Given that the constructed regression models did not explain interindividual differences better than age and gender alone, we conclude that both tasks did not provide incremental information on self-reported differences. Importantly, various explanations are conceivable for this lack of findings. For once, it is possible that self-reported interindividual differences had low convergence with task outcomes due to measuring different aspects of approach-avoidance tendencies or being not suited to assess facets of approach-avoidance tendencies in general. We find the latter unlikely as self-reported interindividual differences had mostly sufficient reliabilities and in part related to the AACT in ways that could be plausibly interpreted in terms of approach-avoidance. Another reason could be the so-called “reliability paradox”: Hedge et al^52^. investigated seven classical cognitive tasks and reported “surprisingly low” reliabilities^52^, p. 1166. They propose that experimental tasks become popular due to their ability to create replicable, homogenous intraindividual task effects across participants, which is achieved by keeping interindividual variability low. However, this reduced interindividual variability makes it harder to achieve robust correlations with external measures such as self-reported questionnaire scores. The visual joystick and manikin tasks have both been designed to elicit reliable differences between approach and avoidance of a particular stimulus category (and especially in contrast to another stimulus category), which might therefore make them less suited for correlational studies which rely on interindividual variability. This interpretation is in line with the subpar split-half consistencies for the bias measures of both tasks. These low reliability estimates hamper correlations of task variables with external measures. For this reason, Goodhew and Edwards^53^ suggested that if studying interindividual differences with experimental tasks, researchers should include accounts of reliability for all measures. We agree that this practice should become commonplace in approach-avoidance research as it may aid understanding under which circumstances interindividual differences can and cannot be measured in approach-avoidance tasks^13^.

In this context, another recommendation from Goodhew and Edwards^53^ should be considered, as they suggested that task conditions or versions should be used that lead to the greatest interindividual variation within task outcome measures. It is possible that our task design, for example, the decision to use many blocks of switching instructions with few stimuli each or the usage of grayscaled happy and angry faces as in prior studies^54,55^ may have reduced interindividual variation. To find task versions and stimuli that are optimal in interindividual differences research, consequences of different task design choices have to be investigated systematically and should be critically reconsidered in future studies. The same is true for the selection of outcome measures. Here, we did not find any meaningful differences between bias scores and D-Scores, but the choice of outcome measure may nonetheless be highly relevant. For example, difference scores, i.e., approach-avoidance biases and D-measures, have generally less reliability than the underlying individual measures, i.e., separate approach and avoidance scores, as measurement error is added up and between-participant variability reduced^52^. Individual measures or other scoring procedures may therefore be more advantageous when researching interindividual differences with approach-avoidance tasks.

### The approach-avoidance conflict task: robust outcome measures relate to behavioral inhibition and physical aggression

In contrast to the visual joystick and manikin tasks, AACT outcome measures related to self-reported behavioral inhibition, physical aggression and verbal aggression, forming clusters of correlations (with uncorrected probabilities) that appear more in line with theoretical predictions. Physical and verbal aggression led to a riskier, but successful strategy (more collected tokens overall) that involved the collection of tokens in the middle of the field, further away from the safe place. For individuals with higher behavioral inhibition scores, the opposite was true. These findings are in line with a prior reporting of cautiousness and daringness correlating with AACT performance^30^. Further, as the BIS is proposed to be sensitive to uncertainty in Gray’s reinforcement sensitivity theory, a stronger relation of the BIS to the AACT with its looming threat compared to the visual joystick and manikin tasks in which the results of one’s own behavior are more certain, i.e., either a zooming in or out of the stimulus or a short clip of the manikin moving towards/away from the stimulus, appears theoretically plausible. Why the other self-reported interindividual differences (behavioral approach, state/trait anxiety, anger and depressive symptoms) did not correlate with task outcomes is unclear given theoretical expectations and comparable internal consistencies (see Table 1). Especially anxiety measures were expected to correlate with the AACT as the task has been validated with anxiolytics in the past, showing that the intake of anxiolytics compared to a placebo led to less anxious behavior, i.e., the participants spending less time in the safe areas^47,56^. Considering that trait anxiety and BIS are both conceptually strongly related as detailed in the introduction, it is surprising that only CW-BIS (and not STAI-T) correlated with AACT behavior. It is possible that this is due to both questionnaires measuring different aspects of (trait) anxiety, for example more items related to anxious apprehension in the CW-BIS and more items related to (the absence of) anxious arousal or anhedonia in the STAI-T. It is therefore possible that a subscore of selected STAI-T items may have been more informative and that content analyses of questionnaires at item-level can be a promising next step to improve associations between traits and specific experimental measures in general^57^. Moreover, it has been discussed that the STAI-T might not be a specific measure of anxiety per se, but rather a non-specific measure of tendency for negative affect^58^. It could thus be discussed that the CW-BIS may be a stronger measure of trait anxiety as the output of the BIS, which would be in line with the pharmacologically validated sensitivity of the AACT towards anxiety^47,56^. Although the reported correlations with uncorrected probabilities (displayed in Fig. 3) should be interpreted with caution, especially since variance might partially be explained by gender differences^30,46^, some of them would remain significant even if conservative comparison corrections had been applied. Additionally, regression analyses that controlled for gender resulted in models including task outcomes as significant predictors for behavioral approach, physical aggression and depressive symptoms.

Despite its advantages, the AACT presents a different challenge in that it offers numerous possible outcome parameters. Here, we selected ten outcome measures based on prior studies^29,56^, some of which shared substantial variance with one another (see Supplementary Table S1). Notably, Bach et al^30^. included 38 outcome parameters in their analyses, illustrating the potential complexity of parameter selection. Consequently, one of the primary challenges for the AACT lies in identifying the most promising parameters, which may depend on the specific interindividual differences being investigated and warrants further exploration in future studies. Furthermore, it is essential to gain better insight into which aspects of approach-avoidance conflicts are related to which outcome parameters, even if this might be difficult as the task scenario is ambiguous and approach and avoidance not clearly separable by design. Differentiating trials based on threat level and initial threat distance (see supplementary section Approach-Avoidance Conflict Task analyses including threat level and threat distance as variables) as well as not only tracing averaged in-trial measures, but looking at their dynamics over time might aid in these efforts^30^. Moreover, unlike the other tasks, which can be easily customized by selecting relevant stimuli for different scenarios, the AACT lacks this adaptability, limiting its scope. Despite these challenges, the AACT appears to be a promising task to measure interindividual differences in approach-avoidance tendencies, which is further supported by the good to excellent reliability of its task outcome measures. In the future, it might be interesting to see how the AACT compares to other types of foraging tasks, for example by Kolling et al^59^., and whether these foraging tasks are more similar in the elicitation of goal-conflict. Direct comparisons with the reaction-time-based tasks presented here were not possible due to an inadequate Kaiser–Meyer–Olkin factor, indicative of the three tasks’ outcome measures not being suited for shared factor analyses in this sample.

### Differences in goal conflict elicitation and further task design choices

Differences between the three tasks, especially the AACT in comparison to the visual joystick and manikin tasks, should be briefly addressed to gain more insight into the different results. We have already discussed some conjectures in the above sections, for example, that the visual joystick and manikin tasks were developed to induce a main congruency effect which may come at the cost of being less reliable in tracking interindividual differences. In addition, it is likely that the tasks differ in their potential to elicit goal-conflicts because of conceptual differences, i.e., visual joystick and manikin tasks measuring automatic approach-avoidance tendencies and the AACT inducing more explicit approach-avoidance conflicts. The tasks differing on the dimension of implicit vs. explicit may be one contributing factor to our findings. Self-report measures are inherently explicit and would therefore be expected to yield higher correlations with other explicit measures compared to more automatic measures due to overlapping response modalities, possibly giving the visual joystick and manikin task a slight disadvantage to find the expected associations. Further, in the visual joystick and manikin tasks, congruent and incongruent actions were manipulated in a block-wise design. While the frequent switching between congruency (16 blocks of 12 trials each) and the zooming effect (visual joystick task)/manikin motion after the button press (manikin task) should have strengthened the elicitation of approach-avoidance conflicts, it is possible that this was in part prevented by proactive or strategic mechanisms of action regulation and cognitive control that reduced conflict by reframing it^60–62^. A reduced goal-conflict would in turn lead to less activation of the BIS, i.e., generate less anxiety, which in turn could reduce associations with self-reported BIS and anxiety measures. To increase goal-conflict in future research, tasks could include stimulus-irrelevant instructions, e.g., to approach all landscape format stimuli and avoid all portrait format stimuli, as this would allow presenting congruent and incongruent trials in random succession. However, it should also be considered that these more implicit instructions may lead to reduced goal-conflicts, if stimuli are largely processed based on features irrelevant to the research question. In the review by Fricke and Vogel, it remained unclear whether stimulus-relevant or -irrelevant task versions were more predictive of interindividual differences as both types of tasks resulted in heterogeneous findings^13^. The AACT, in contrast, induces approach-avoidance conflicts which likely varied in strength between trials due to differences in threat level and initial threat distance as well as experiences in prior trials. This stronger induction of goal-conflict may therefore be one reason why the AACT related to the self-report measures investigated here.

## Conclusion

In conclusion, our findings indicate that the visual joystick and manikin task measures are not comparable operationalizations of the same underlying constructs and have limited associations with the self-reported interindividual differences examined in this study. This is noteworthy considering our sufficiently large and heterogeneous sample, the identical structure of both tasks, the use of similar stimuli, and the application of identical analysis procedures. Furthermore, the approach-avoidance bias measures in both tasks showed subpar reliability. Conversely, the AACT seems to be associated with several interindividual differences and demonstrated good to excellent split-half reliabilities.

Our study highlights the need for further research to determine the most promising task-based measures of approach and avoidance tendencies when investigating interindividual variability. Besides improving prediction (of individual differences), we would like to emphasize that our findings also have repercussions for much-needed studies using experimental approaches to better understand the precise mechanisms governing approach avoidance behaviors. For instance, for both reaction-time-based tasks, reliabilities should consistently be reported per study and efforts should be made to improve them. Stimulus selection may be especially of relevance as higher reliabilities were achieved with different stimulus sets (images of spiders)^8,12^. Stimulus saliency may also be increased, for example, by testing approach-avoidance tendencies in more immersive and ecologically valid virtual reality settings^63^. As the tasks do not appear to measure the same construct, it might be of interest to further investigate why the tasks differ from one another. Alternative tasks such as the AACT might be advantageous, but have their own shortcomings such as potentially more rigid designs and, in most cases, limited literature to support their efficacy. To effectively measure individual differences in approach-avoidance tendencies operationalized by behavioral tasks with high criterion validity, careful consideration of task selection, stimulus materials, and ensuring sufficient reliability will be required.

### Supplementary Information

Supplementary Information.

## Data Availability

All analyses were conducted in R (Version 4.3.1) and can be found at osf.io/ahvzx. The data is made available at osf.io/ahvzx within the analysis structure. The images used in the visual joystick and manikin task can be requested from kdef.se and rafd.socsci.ru.nl/RaFD2/RaFD for non-commercial scientific research purposes.
