# Supplementary material for: Comparison of two reaction-time-based and one foraging-based behavioral approach-avoidance tasks in relation to interindividual differences and their reliability
Source: Sci Rep. 2023 Dec 16;13:22376. doi: 10.1038/s41598-023-49864-x (PMC10725419; doi:10.1038/s41598-023-49864-x)
Supplement: Supplementary file 1 — Supplementary Information. [file 41598_2023_49864_MOESM1_ESM.pdf]

Manikin Task Stimulus Set (from KDEF [1] and Radboud Faces Databank [2])

Angry Male Faces	Angry Female Faces	Happy Male Faces	Happy Female Faces
AM17ANS	BF11ANS	AM04HAS	AF20HAS
AM28ANS	AF13ANS	AM16HAS	AF24HAS
BM05ANS	BF15ANS	AM23HAS	AF26HAS
BM15ANS	BF20ANS	BM10HAS	BF06HAS
BM17ANS	BF21ANS	BM20HAS	BF07HAS
BM25ANS	BF24ANS	BM22HAS	AF11HAS
BM27ANS	BF33ANS	BM32HAS	BF24HAS
BM28ANS	Rafd090_01_Caucasian_female_angry_frontal	Rafd090_07_Caucasian_male_happy_frontal	Rafd090_14_Caucasian_female_happy_frontal
BM34ANS	Rafd090_61_Caucasian_female_angry_frontal	Rafd090_25_Caucasian_male_happy_frontal	Rafd090_56_Caucasian_female_happy_frontal
Rafd090_05_Caucasian_male_angry_frontal	AF01ANS	Rafd090_33_Caucasian_male_happy_frontal	AF02HAS
Rafd090_23_Caucasian_male_angry_frontal	AF05ANS	Rafd090_46_Caucasian_male_happy_frontal	AF15HAS
Rafd090_28_Caucasian_male_angry_frontal	AF14ANS	Rafd090_71_Caucasian_male_happy_frontal	AF17HAS

Visual Joystick Task Stimulus Set (from KDEF and Radboud Faces Databank)

Angry Male Faces	Angry Female Faces	Happy Male Faces	Happy Female Faces
AM09ANS	AF21ANS	AM05HAS	AF19HAS
AM29ANS	AF25ANS	AM22HAS	AF22HAS
AM32ANS	AF33ANS	AM24HAS	AF33HAS
BM01ANS	BF01ANS	AM32HAS	BF02HAS
BM03ANS	BF16ANS	AM34HAS	BF17HAS
BM06ANS	BF17ANS	BM07HAS	BF22HAS
BM07ANS	BF29ANS	BM16HAS	AF27HAS
BM09ANS	Rafd090_16_Caucasian_female_angry_frontal	BM23HAS	BF30HAS
BM10ANS	AF07ANS	BM25HAS	BF31HAS
BM16ANS	AF13ANS	Rafd090_10_Caucasian_male_happy_frontal	BF33HAS
BM23ANS	AF16ANS	Rafd090_21_Caucasian_male_happy_frontal	Rafd090_04_Caucasian_female_happy_frontal
BM29ANS	AF17ANS	Rafd090_30_Caucasian_male_happy_frontal	BF11HAS

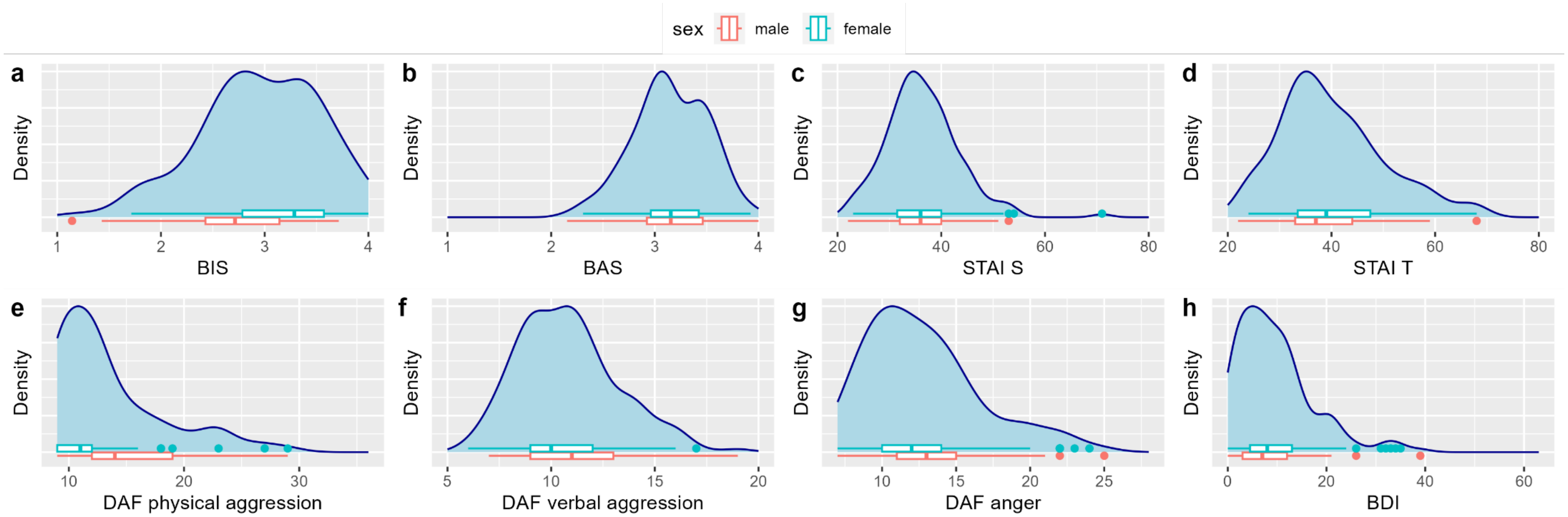

Supplemental Figure S1. Density plots of (a) BIS, (b) BAS, (c) STAI S, (d) STAI T, (e) physical aggression, (f) verbal aggression, (g) anger, and (h) BDI scores with box-plots separated by gender.

Supplemental Table S1. *Approach avoidance conflict task outcome measure correlations with confidence intervals*

Variable	1	2	3	4	5	6	7	8	9
1. Presence in safe quadrant									
2. Presence in safe place	.49**<br>[.37, .60]								
3. Distance to closest wall	-.72**<br>[-.78, -.64]	-.71**<br>[-.78, -.63]							
4. Speed on grid	-.40**<br>[-.52, -.27]	-.40**<br>[-.52, -.26]	.64**<br>[.54, .72]						
5. Presence in dangerous quadrant	-.77**<br>[-.83, -.70]	-.23**<br>[-.37, -.09]	.42**<br>[.28, .54]	.05<br>[-.11, .20]					
6. Distance to predator	.97**<br>[.96, .98]	.53**<br>[.41, .63]	-.71**<br>[-.78, -.63]	-.32**<br>[-.45, -.18]	-.86**<br>[-.90, -.82]				
7. Token collection rate	-.56**<br>[-.66, -.45]	-.52**<br>[-.62, -.40]	.81**<br>[.75, .86]	.81**<br>[.75, .86]	.22**<br>[.07, .36]	-.50**<br>[-.61, -.38]			
8. Collected Tokens	-.23**<br>[-.37, -.08]	-.36**<br>[-.49, -.22]	.62**<br>[.51, .70]	.79**<br>[.72, .84]	-.09<br>[-.24, .06]	-.16*<br>[-.31, -.01]	.91**<br>[.88, .93]		
9. Foraging latency	.16*<br>[.01, .31]	.31**<br>[.16, .44]	-.46**<br>[-.57, -.34]	-.67**<br>[-.74, -.57]	.06<br>[-.09, .21]	.10<br>[-.06, .24]	-.57**<br>[-.67, -.46]	-.61**<br>[-.70, -.51]	
10. Player caught rate	-.82**<br>[-.87, -.77]	-.46**<br>[-.57, -.33]	.52**<br>[.40, .62]	.08<br>[-.07, .23]	.74**<br>[.67, .81]	-.86**<br>[-.89, -.81]	.23**<br>[.08, .37]	-.19*<br>[-.33, -.04]	.10<br>[-.05, .25]

Note. Values in square brackets indicate the 95% confidence interval for each correlation. The confidence interval is a plausible range of population correlations that could have caused the sample correlation (Cumming, 2014). * indicates $p < .05$. ** indicates $p < .01$.

Approach-Avoidance Conflict Task analyses including threat level and threat distance as variables

To investigate potential influences of threat level and initial threat distance on the association between outcome parameters in the AACT and self-reported individual differences (see article), all ten parameters were split by threat level and initial threat distance, leading to 4x10 outcome parameters. These parameters were then subjected to the same analyses described for Aim 2 in the analysis section of the main manuscript. It is important to note that due to the process of the lasso regression variable selection, interpretations based on the data presented in the regression models (see Supplement-Specific Table SS1) should be made carefully and with the strong intercorrelation of AACT variables in mind (see also Supplemental Table S1). The correlations indicate that an increase in data resolution is especially relevant for threat distance as correlations are often found for either far or close initial threat distance (see Supplement-Specific Figure SS1 and Supplement-Specific Table SS2). Surprisingly, self-reported BIS did not seem to depend on the specific threat configuration, which might have been expected since the experienced approach-avoidance conflict depends on differences in initial threat distance and threat level. In the future, researcher could consider to also introduce an intermediate distance of threat, in which participants are placed in the middle of the field initially or find other ways to manipulate threat distance and threat level further.

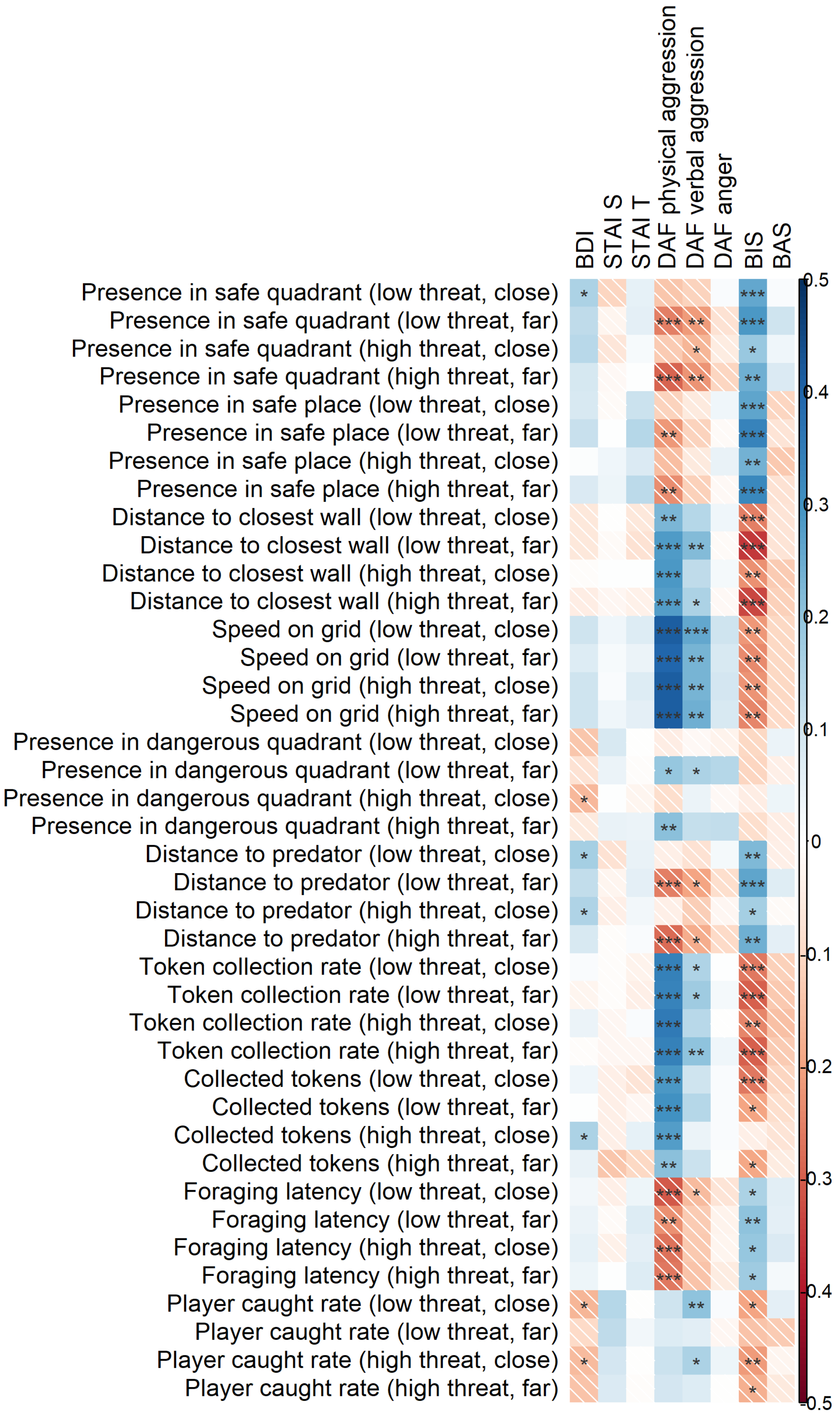

*Supplement-specific Figure SS1. Correlation matrix of all questionnaire scores with AACT task outcome measures split by threat level (low/ high threat) and initial threat distance (close, far). Color intensity indicates strength of correlation, color and shade indicate direction of effect (blue/no shade = positively correlated; red/ shaded = negatively correlated). p-values are uncorrected for multiple comparisons. Significance codes: *** p < .001, ** p < .01, * p < .05.*

Supplement-specific Table SS1. Significantly better multiple regression models including threat level and initial threat distance of the AACT compared to baseline models including only age and gender (with predictors)

		Original Model	Alternative Model
Questionnaire	Predictor Variables	Model fit / sig. β estimates	Model fit / sig. β estimates
BIS			adj. R^2 = .19, $F(6,156)$ = 6.568, p <.001
	**Gender**		0.310**
	Age		.
	Collected Tokens (low threat, close)		.
	Presence in safe quadrant (low threat, close)		.
	Presence in safe place (low threat, far)		
	Presence in safe place (high threat, far)		
	Distance to closest wall (low threat, far)		.
BAS		adj. R^2 = .10, $F(9,158)$ = 2.986, p =.003	adj. R^2 = .13, $F(9,152)$ = 3.656, p <.001
	Gender	.	.
	Age	.	.
	Player caught rate (low threat, close)	.	.
	**Player caught rate (low threat, far)**	-0.416**	-0.342*
	Distance to predator (low threat, close)		
	Presence in safe place (low threat, far)	.	
	**Presence in safe place (high threat, close)**	.	-2.915*
	**Distance to closest wall (high threat, close)**	.	-0.315*
	**Token Collection Rate (high threat, close)**	.	-1.205*
verbal aggression		adj. R^2 = .08, $F(5,162)$ = 3.876, p =.002	adj. R^2 = .10, $F(5,158)$ =4.494, p <.001
	Gender		.
	Age		.
	**Player caught rate (low threat, close)**	1.921*	
	**Speed on grid (low threat, close)**	1.340*[a]	1.458*
	Presence in safe quadrant (high threat, far)		.
BDI		adj. R^2 = .10, $F(10,155)$ = 2.788, p =.003	adj. R^2 = .07, $F(10,147)$ = 2.650, p =.018
	**Gender**	3.447*[a]	.
	Age	.	.
	Player caught rate (low threat, close)	.	.
	Distance to predator (low threat, far)		
	Presence in safe quadrant (low threat, far)		
	Presence in safe place (low threat, far)		
	Collected Tokens (high threat, close)		
	**Foraging Latency (high threat, close)**	0.008*	.
	Presence in dangerous quadrant (high threat, close)	.	.
	**Speed on grid (high threat, far)**	7.304**	5.759**

Notes. Alternative model: Model without cases with standardized residuals exceeding two standard deviations away from the mean and Cook's distance exceeding a value of one, leverage values three times larger than the average leverage and/or a covariance ratio outside of one plus/minus three times the average leverage. Low/high threat stands for the respective threat level, while far and close represent the initial threat distance. Significant predictors are in bold. [a]Not significant based on bootstrapped confidence interval. Significance codes: *** $p < 0.001$, ** $p < 0.01$, * $p < 0.05$

Supplement-specific Table SS2. Correlations of AACT outcome measures including threat level and initial threat distance with self-report questionnaire scores

	BDI TOTAL	STAI S	STAI T	PHYSICAL AGGRESSION	VERBAL AGGRESSION	ANGER	BIS	BAS
**PRESENCE IN SAFE QUADRANT (LOW THREAT, CLOSE)**	r = 0.16, p = 0.04	r = -0.11, p = 0.157	r = 0.05, p = 0.509	r = -0.14, p = 0.08	r = -0.11, p = 0.141	r = 0.01, p = 0.862	r = 0.26, p = 0.001	r = 0.01, p = 0.855
**PRESENCE IN SAFE QUADRANT (LOW THREAT, FAR)**	r = 0.13, p = 0.105	r = -0.03, p = 0.727	r = 0.06, p = 0.469	r = -0.25, p = 0.001	r = -0.21, p = 0.006	r = -0.08, p = 0.307	r = 0.29, p < 0.001	r = 0.1, p = 0.188
**PRESENCE IN SAFE QUADRANT (HIGH THREAT, CLOSE)**	r = 0.14, p = 0.072	r = -0.07, p = 0.386	r = 0.02, p = 0.827	r = -0.13, p = 0.106	r = -0.17, p = 0.031	r = -0.05, p = 0.48	r = 0.19, p = 0.014	r = 0.03, p = 0.662
**PRESENCE IN SAFE QUADRANT (HIGH THREAT, FAR)**	r = 0.09, p = 0.265	r = -0.02, p = 0.82	r = 0, p = 0.977	r = -0.29, p < 0.001	r = -0.22, p = 0.004	r = -0.11, p = 0.159	r = 0.24, p = 0.001	r = 0.08, p = 0.33
**PRESENCE IN SAFE PLACE (LOW THREAT, CLOSE)**	r = 0.08, p = 0.291	r = -0.01, p = 0.896	r = 0.11, p = 0.175	r = -0.11, p = 0.141	r = -0.06, p = 0.462	r = 0.03, p = 0.696	r = 0.26, p = 0.001	r = -0.11, p = 0.172
**PRESENCE IN SAFE PLACE (LOW THREAT, FAR)**	r = 0.11, p = 0.153	r = 0, p = 0.99	r = 0.14, p = 0.065	r = -0.22, p = 0.004	r = -0.11, p = 0.153	r = -0.01, p = 0.89	r = 0.33, p < 0.001	r = -0.07, p = 0.342
**PRESENCE IN SAFE PLACE (HIGH THREAT, CLOSE)**	r = 0.01, p = 0.933	r = 0.03, p = 0.67	r = 0.08, p = 0.324	r = -0.15, p = 0.052	r = -0.06, p = 0.469	r = 0.05, p = 0.555	r = 0.24, p = 0.002	r = -0.13, p = 0.092
**PRESENCE IN SAFE PLACE (HIGH THREAT, FAR)**	r = 0.08, p = 0.315	r = 0.04, p = 0.634	r = 0.13, p = 0.092	r = -0.23, p = 0.003	r = -0.12, p = 0.128	r = -0.02, p = 0.822	r = 0.32, p < 0.001	r = -0.08, p = 0.304
**DISTANCE TO CLOSEST WALL (LOW THREAT, CLOSE)**	r = -0.06, p = 0.436	r = 0, p = 0.977	r = -0.06, p = 0.425	r = 0.23, p = 0.003	r = 0.14, p = 0.064	r = 0.03, p = 0.694	r = -0.25, p = 0.001	r = -0.07, p = 0.345
**DISTANCE TO CLOSEST WALL (LOW THREAT, FAR)**	r = -0.06, p = 0.412	r = -0.01, p = 0.849	r = -0.08, p = 0.327	r = 0.28, p < 0.001	r = 0.22, p = 0.004	r = -0.01, p = 0.885	r = -0.35, p < 0.001	r = -0.07, p = 0.366
**DISTANCE TO CLOSEST WALL (HIGH THREAT, CLOSE)**	r = -0.01, p = 0.908	r = 0, p = 0.974	r = 0, p = 0.984	r = 0.29, p < 0.001	r = 0.13, p = 0.106	r = 0.02, p = 0.779	r = -0.23, p = 0.003	r = -0.13, p = 0.099
**DISTANCE TO CLOSEST WALL (HIGH THREAT, FAR)**	r = -0.05, p = 0.547	r = -0.02, p = 0.776	r = -0.04, p = 0.65	r = 0.28, p < 0.001	r = 0.16, p = 0.042	r = -0.02, p = 0.816	r = -0.33, p < 0.001	r = -0.12, p = 0.131
**SPEED ON GRID (LOW THREAT, CLOSE)**	r = 0.1, p = 0.181	r = 0.03, p = 0.693	r = 0.07, p = 0.36	r = 0.41, p < 0.001	r = 0.26, p = 0.001	r = 0.1, p = 0.187	r = -0.22, p = 0.004	r = -0.1, p = 0.184
**SPEED ON GRID (LOW THREAT, FAR)**	r = 0.07, p = 0.344	r = 0.02, p = 0.812	r = 0.04, p = 0.57	r = 0.4, p < 0.001	r = 0.23, p = 0.002	r = 0.08, p = 0.28	r = -0.24, p = 0.002	r = -0.1, p = 0.188
**SPEED ON GRID (HIGH THREAT, CLOSE)**	r = 0.1, p = 0.198	r = 0.01, p = 0.865	r = 0.07, p = 0.362	r = 0.41, p < 0.001	r = 0.23, p = 0.002	r = 0.09, p = 0.242	r = -0.22, p = 0.004	r = -0.1, p = 0.187
**SPEED ON GRID (HIGH THREAT, FAR)**	r = 0.1, p = 0.192	r = 0.03, p = 0.678	r = 0.06, p = 0.476	r = 0.41, p < 0.001	r = 0.24, p = 0.001	r = 0.08, p = 0.288	r = -0.24, p = 0.001	r = -0.1, p = 0.211
**PRESENCE IN DANGEROUS QUADRANT (LOW THREAT, CLOSE)**	r = -0.14, p = 0.072	r = 0.08, p = 0.3	r = 0, p = 0.965	r = -0.05, p = 0.55	r = -0.02, p = 0.817	r = -0.03, p = 0.674	r = -0.1, p = 0.178	r = 0.04, p = 0.573
**PRESENCE IN DANGEROUS QUADRANT (LOW THREAT, FAR)**	r = -0.08, p = 0.32	r = 0.04, p = 0.576	r = -0.01, p = 0.925	r = 0.19, p = 0.012	r = 0.16, p = 0.038	r = 0.14, p = 0.065	r = -0.11, p = 0.157	r = -0.04, p = 0.581
**PRESENCE IN DANGEROUS QUADRANT (HIGH THREAT, CLOSE)**	r = -0.16, p = 0.036	r = 0, p = 0.973	r = -0.03, p = 0.738	r = -0.09, p = 0.258	r = 0.04, p = 0.577	r = -0.02, p = 0.84	r = -0.05, p = 0.553	r = 0.04, p = 0.627
**PRESENCE IN DANGEROUS QUADRANT (HIGH THREAT, FAR)**	r = -0.06, p = 0.451	r = 0.05, p = 0.554	r = 0.04, p = 0.574	r = 0.21, p = 0.007	r = 0.12, p = 0.124	r = 0.12, p = 0.118	r = -0.09, p = 0.272	r = -0.05, p = 0.554
**DISTANCE TO PREDATOR (LOW THREAT, CLOSE)**	r = 0.17, p = 0.031	r = -0.08, p = 0.325	r = 0.05, p = 0.55	r = -0.04, p = 0.596	r = -0.08, p = 0.313	r = 0.02, p = 0.782	r = 0.22, p = 0.004	r = -0.04, p = 0.597
**DISTANCE TO PREDATOR (LOW THREAT, FAR)**	r = 0.12, p = 0.122	r = -0.03, p = 0.738	r = 0.06, p = 0.471	r = -0.25, p = 0.001	r = -0.2, p = 0.01	r = -0.09, p = 0.247	r = 0.26, p = 0.001	r = 0.07, p = 0.389
**DISTANCE TO PREDATOR (HIGH THREAT, CLOSE)**	r = 0.15, p = 0.05	r = -0.04, p = 0.593	r = 0.03, p = 0.725	r = -0.04, p = 0.593	r = -0.12, p = 0.11	r = -0.02, p = 0.754	r = 0.17, p = 0.028	r = -0.01, p = 0.851
**DISTANCE TO PREDATOR (HIGH THREAT, FAR)**	r = 0.08, p = 0.293	r = -0.01, p = 0.901	r = 0.01, p = 0.872	r = -0.28, p < 0.001	r = -0.18, p = 0.019	r = -0.1, p = 0.215	r = 0.24, p = 0.002	r = 0.06, p = 0.446
**TOKEN COLLECTION RATE (LOW THREAT, CLOSE)**	r = 0.01, p = 0.877	r = -0.01, p = 0.932	r = -0.03, p = 0.696	r = 0.34, p < 0.001	r = 0.15, p = 0.048	r = 0, p = 0.994	r = -0.26, p = 0.001	r = -0.12, p = 0.125
**TOKEN COLLECTION RATE (LOW THREAT, FAR)**	r = -0.03, p = 0.743	r = -0.01, p = 0.914	r = -0.04, p = 0.588	r = 0.33, p < 0.001	r = 0.18, p = 0.023	r = 0.02, p = 0.785	r = -0.3, p < 0.001	r = -0.13, p = 0.087
**TOKEN COLLECTION RATE (HIGH THREAT, CLOSE)**	r = 0.04, p = 0.608	r = -0.02, p = 0.789	r = 0.01, p = 0.848	r = 0.35, p < 0.001	r = 0.14, p = 0.073	r = 0, p = 0.999	r = -0.24, p = 0.002	r = -0.15, p = 0.055
**TOKEN COLLECTION RATE (HIGH THREAT, FAR)**	r = -0.01, p = 0.916	r = -0.02, p = 0.775	r = -0.02, p = 0.779	r = 0.34, p < 0.001	r = 0.2, p = 0.009	r = 0.03, p = 0.676	r = -0.3, p < 0.001	r = -0.13, p = 0.104
**COLLECTED TOKENS (LOW THREAT, CLOSE)**	r = 0.03, p = 0.687	r = -0.04, p = 0.566	r = -0.07, p = 0.341	r = 0.29, p < 0.001	r = 0.1, p = 0.18	r = 0.01, p = 0.878	r = -0.26, p = 0.001	r = -0.11, p = 0.172
**COLLECTED TOKENS (LOW THREAT, FAR)**	r = 0, p = 0.953	r = -0.04, p = 0.597	r = -0.02, p = 0.794	r = 0.3, p < 0.001	r = 0.14, p = 0.062	r = 0.01, p = 0.893	r = -0.2, p = 0.011	r = -0.09, p = 0.248

**COLLECTED TOKENS (HIGH THREAT, CLOSE)**	r = 0.16, p = 0.045	r = -0.04, p = 0.566	r = 0.05, p = 0.489	r = 0.28, p < 0.001	r = 0.04, p = 0.566	r = 0.01, p = 0.862	r = -0.04, p = 0.568	r = -0.07, p = 0.346
**COLLECTED TOKENS (HIGH THREAT, FAR)**	r = 0.05, p = 0.562	r = -0.14, p = 0.076	r = -0.1, p = 0.178	r = 0.21, p = 0.007	r = 0.11, p = 0.172	r = 0.01, p = 0.91	r = -0.19, p = 0.012	r = -0.05, p = 0.498
**FORAGING LATENCY (LOW THREAT, CLOSE)**	r = 0.03, p = 0.715	r = -0.04, p = 0.598	r = 0.04, p = 0.62	r = -0.31, p < 0.001	r = -0.16, p = 0.043	r = -0.07, p = 0.336	r = 0.16, p = 0.04	r = 0.06, p = 0.418
**FORAGING LATENCY (LOW THREAT, FAR)**	r = 0.04, p = 0.571	r = -0.01, p = 0.862	r = 0.07, p = 0.336	r = -0.23, p = 0.003	r = -0.13, p = 0.095	r = -0.03, p = 0.678	r = 0.2, p = 0.009	r = 0.06, p = 0.448
**FORAGING LATENCY (HIGH THREAT, CLOSE)**	r = 0.05, p = 0.492	r = -0.04, p = 0.631	r = 0.06, p = 0.464	r = -0.27, p < 0.001	r = -0.13, p = 0.089	r = -0.03, p = 0.705	r = 0.19, p = 0.012	r = 0.08, p = 0.325
**FORAGING LATENCY (HIGH THREAT, FAR)**	r = 0.04, p = 0.611	r = 0, p = 0.983	r = 0.07, p = 0.367	r = -0.26, p = 0.001	r = -0.14, p = 0.062	r = -0.05, p = 0.485	r = 0.18, p = 0.023	r = 0.02, p = 0.762
**PLAYER CAUGHT RATE (LOW THREAT, CLOSE)**	r = -0.17, p = 0.033	r = 0.14, p = 0.061	r = 0, p = 0.969	r = 0.1, p = 0.196	r = 0.2, p = 0.008	r = 0.01, p = 0.856	r = -0.2, p = 0.01	r = 0.06, p = 0.452
**PLAYER CAUGHT RATE (LOW THREAT, FAR)**	r = -0.1, p = 0.209	r = 0.13, p = 0.096	r = 0.03, p = 0.714	r = 0.07, p = 0.339	r = 0.06, p = 0.419	r = -0.02, p = 0.785	r = -0.14, p = 0.067	r = -0.13, p = 0.094
**PLAYER CAUGHT RATE (HIGH THREAT, CLOSE)**	r = -0.16, p = 0.042	r = 0.09, p = 0.231	r = 0, p = 0.959	r = 0.11, p = 0.167	r = 0.16, p = 0.042	r = 0.04, p = 0.628	r = -0.21, p = 0.006	r = -0.03, p = 0.743
**PLAYER CAUGHT RATE (HIGH THREAT, FAR)**	r = -0.14, p = 0.066	r = 0.08, p = 0.305	r = -0.01, p = 0.932	r = 0.09, p = 0.227	r = 0.07, p = 0.363	r = 0, p = 0.981	r = -0.18, p = 0.021	r = -0.06, p = 0.474

References

1 Lundqvist D, Flykt A, Öhman A. (1998).

2 Langner O, Dotsch R, Bijlstra G, Wigboldus DHJ, Hawk ST, van Knippenberg A. Presentation and validation of the Radboud Faces Database. Cognition & Emotion. 2010;24(8):1377-88.
